# Evaluation of Environmentally Relevant Nitrated and Oxygenated Polycyclic Aromatic Hydrocarbons in Honey

**DOI:** 10.3390/foods12112205

**Published:** 2023-05-31

**Authors:** Alejandro Mandelli, María Guiñez, Soledad Cerutti

**Affiliations:** 1 Mass Spectrometry Lab, Chemistry Department, Faculty of Chemistry, Biochemistry and Pharmacy, Institute of Chemistry of San Luis (INQUISAL, UNSL-CONICET CCT-San Luis), National University of San Luis, Block III, 950 Ejercito de los Andes, San Luis D5700 HHW, Argentina; alemandelli87@gmail.com (A.M.); maevangelinaguinez@gmail.com (M.G.); 2 National Council of Scientific and Technical Research (CONICET), 2290 Godoy Cruz, Buenos Aires C1425 FQB, Argentina

**Keywords:** nitrated and oxygenated polycyclic aromatic hydrocarbons, bee honey, food quality control, sustainability assessment

## Abstract

In this work, a novel analytical methodology for the extraction and determination of polycyclic aromatic hydrocarbon derivatives, nitrated (NPAH) and oxygenated (OPAH), in bee honey samples was developed. The extraction approach resulted in being straightforward, sustainable, and low-cost. It was based on a salting-out assisted liquid-liquid extraction followed by liquid chromatography-tandem mass spectrometry determination (SALLE-UHPLC-(+)APCI-MS/MS). The following figures of merit were obtained, linearity between 0.8 and 500 ng g^−1^ for NPAH and between 0.1 and 750 ng g^−1^ for OPAH compounds, coefficients of determination (r2) from 0.97 to 0.99. Limits of detection (LOD) were from 0.26 to 7.42 ng g^−1^ for NPAH compounds and from 0.04 to 9.77 ng g^−1^ for OPAH compounds. Recoveries ranged from 90.6% to 100.1%, and relative standard deviations (RSD) were lower than 8.9%. The green assessment of the method was calculated. Thus, the Green Certificate allowed a classification of 87 points. This methodology was reliable and suitable for application in honey samples. The results demonstrated that the levels of nitro- and oxy-PAHs were higher than those reported for unsubstituted PAHs. In this sense, the production chain sometimes transforms foods as direct carriers of contaminants to consumers, representing a concern and demonstrating the need for routine control.

## 1. Introduction

Pollution comprises any substance in water, soil, or air that degrades the natural quality of the environment and creates a health hazard [1]. It is one of the most critical problems in the world, which generates the need and the search for alternatives to mitigate and correct the adverse effects caused in the ecosystems.

Environmental pollutants include compounds released into the environment from natural or anthropogenic sources [2]. The primary emission sources are organic material burning, vehicle exhausts, and industrial and agricultural activities [3,4].

Persistent Organic Pollutants (POP) owe their name to their organic nature. These hazardous chemicals are recognized as a serious global threat to human health and ecosystems [5]. There has been a growing interest in studying polycyclic aromatic hydrocarbons and their derivatives in recent decades. Its importance lies in its varied chemical nature and origin, its presence in the environment, and the possible consequences, which have gone largely unnoticed, causing environmental and health risk problems. These compounds are widely distributed and detected in various environmental compartments, crops, and food samples. According to research on their potential health effects, they are still unregulated pollutants and may be candidates for future control [6].

Nitrated and oxygenated PAH derivatives (NPAH and OPAH, respectively) constitute a subgroup of compounds that have one or more nitro (-NO_2_) or carbonyl (-CO) moieties as substitutes in their structure (the specific PAH derivatives studied in this work are detailed in Section 2.1). Thus, NPAH and OPAH are mainly formed by atmospheric reactions with oxidizing species, such as ozone, hydroxyl, nitrate radicals, and UV-induced photoreactions. These stable PAH oxidation products are typically found in medium polarity fractions and include many compounds. On the other hand, the strong evidence of the high toxicity of nitrated and oxygenated PAH (even more carcinogenic and mutagenic than PAH) has led to a growing interest in chemical analysis, occurrence, fate, and behavior of them in different environmental compartments [6,7,8,9,10,11,12,13,14], and evaluation of their occurrence in food. Exposure to these compounds is associated with acute and chronic dermatitis, symptoms of burning, itching, and edema, being more pronounced in the regions of the exposed skin. In addition, irritation of the upper airways may also be observed. In the eyes, they produce tearing, photophobia, eyelid edema, and conjunctival hyperemia [15]. Prolonged exposure can lead to skin, bronchogenic, and bladder cancer; the hematopoietic system can cause leukemia and lymphoma. The impact of PAH and its derivatives on human health depends on their concentration, route of exposure, and relative toxicity. In this sense, lethal PAH doses are estimated to be between 5 and 15 g day^−1^ in adults and 2 g day^−1^ in children [16,17].

Food intake in non-smokers is the first route of contamination by these toxic compounds. PAH and its derivatives can appear in food during storage, transportation, or cooking processes (mainly grilled, roasted, smoked, and fried). In addition, the use of contaminated water and soil during the stages of washing and receiving raw materials, crop growth, incineration of agricultural waste, and burning of biomass, together with the deposition of air particles in food during post-harvest and processing phases, contribute to the presence of PAH and their drift in food samples [17].

In this sense, honey is a nutritious food that provides immediate energy to the body through the presence of simple sugars that are assimilated. At the same time, it owns the property to inhibit the growth of bacteria and promote recovery in some nutritional conditions and imbalances. It is produced by honeybees from the nectar of flowers, secretions from living parts of plants, and/or excretions of plant suction insects’ leftover living parts of them [18,19,20,21,22]. In addition, this activity has a high social and economic impact [22].

During production, *honey apis bees* make an average of 10 trips covering an area of approximately 13.5 km^2^ around the apiary, collecting nectar, water, and pollen from flowers [23]. During their flight, they can contact, and capture substances suspended in the air that can accumulate in the bee’s body and can be trafficked into hive products. In addition, the location of apiaries near industrial or urban areas and automotive traffic, incorrect handling during honey processing, and the use of agrochemicals and pesticides on the honey flora or in areas close to the hive location may cause their products to present substances outside its composition which may be harmful to the health of consumers.

Considering the complexity of food samples, the variety of chemical structures of the pollutants of interest, and the low concentrations to which they must be detected and quantified, developing methodologies that provide reliable and accurate results is needed.

Consequently, these methods should be fast, simple, safe for analysts, and environmentally friendly, characteristics that decisively stand up to their acceptance in routine analyses. As well known, sample treatment is the most complex and laborious step of the analytical processes commonly used for environmental and food analysis. It usually involves different operations that consume most of the time required and provide an important source of uncertainty in the measurements.

In this sense, the traditional methods for extracting and determining PAH and nitrated and oxygenated derivatives in environmental and food interest samples are liquid-liquid extraction, Soxhlet extraction, ultrasound-assisted or microwave-assisted extraction, solid-phase extraction, and solvent-accelerated extraction [6,24,25,26,27,28]. Due to the compounds’ low concentration (µg g^−1^ or less) and the complexity of these types of samples, the extraction techniques used involve stages of preconcentration and protocols of additional clean-up, making the obtained extracts compatible with the detection system. 

Particularly in liquid-liquid extractions (LLE), the ionic strength effect (salting-out) by adding sodium chloride (or other salts) to the aqueous matrix solution is widely used to increase the ionic strength, decrease the aqueous solubility of the analytes of interest, improving its extraction efficiency. In general, adding electrolytes to the extraction medium allows for counteracting the effects of solvating forces in favor of the extraction solvents. Although the salt saturation extraction technique (SALLE, [29]) has already been applied for food samples, it has not yet been described for the extraction of nitrated and oxygenated PAH derivatives. Furthermore, a lack of information on the analysis of these compounds in honey samples has been observed, and existing studies focus only on the evaluation of PAH. Thus, the analysis of PAH derivatives in honey samples constitutes a novel analytical approach.

The most used techniques for determining PAH and derivatives In food samples (both at trace and higher concentrations) are gas chromatography, with some limitations regarding the volatility and instability of some compounds, and liquid chromatography. Thus, LC-coupled to MS or MS/MS, with interfaces such as atmospheric pressure chemical ionization and electrospray or atmospheric pressure photoionization, achieve high sensitivity, with detection and quantification limits in the order of a few ng kg^−1^.

In any analytical methodology, the sample preparation stage is usually considered the most important, where most of the reagents and inputs are involved and where multiple reagents and solvents are consumed, thus generating (toxic) waste. This fact needs to be more exhaustively considered when highlighting the benefits of the developed methodologies. The first steps towards what is today Green Analytical Chemistry (GAC) were proposed by Guardia and Ruzicka in 1995 [30]. Later, in 1998, Anastas and Warner [31] first established their principles to reduce or eliminate the side effects of analytical practices on operators and the environment. Therefore, a “green analytical procedure” must be able to solve a problem with the highest level of reliability, generating the minimum impact on the environment and the operator. The first approach is the Eco-Analytical Scale [32]; this tool is based on penalty points subtracted from a base of 100. The higher the score, the greener and more economical the analytical procedure. 

In summary, this work proposes a novel and sensitive analytical methodology for accurately determining nitrated and oxygenated PAH of environmental concern in bee honey samples. The sustainability of the proposed methodology was also evaluated, and the results are presented through the Eco-Analytical Scale and the green certificate. This development presents an effective and practical analytical tool for honey quality assessment for the first time.

## 2. Materials and Methods

### 2.1. Chemicals

Chemical standards of nitrated and oxygenated PAH derivatives, 1-nitropyrene (1-NPYR), 2-nitrofluorene (2-NFLU), 3-nitrofluoranthene (3-NFLUANTH), 9-nitroanthracene (9-NANTHR), 9,10-anthracenedione (9,10-ANTHRONE), 5,12-Naphthacenequinone (5,12-NAPHTONA), and 2-fluorenecarboxaldehyde (2-FLUCHO), were purchased from Sigma Chemical (St. Louis, MO, USA) structures and properties are shown in Table S1. Acetonitrile (ACN), methanol (MeOH), and water Optima^®®^ LC-MS grade (Fair Lawn, NJ, USA) and acetone, cyclohexane, toluene, and n-hexane HPLC^®^ grade obtained from Fisher Scientific (Fair Lawn, NJ, USA). Formic acid was purchased from Fisher Scientific (Loughborough, UK). Ultrapure water (18 MΩ cm) from a Milli-Q water purification system from EASY pure (RF Barnstead, Dubuque, IA, USA) was used. Sodium chloride, Potassium chloride, and Ammonium sulfate were purchased from Sigma-Aldrich (St. Louis, MO, USA), and Nitrogen and Argon were purchased from Air Liquide (CABA, Buenos Aires, Argentina). Acetonitrile-based working standard solutions were prepared by stepwise dilution from a 200 μg mL^−1^ standard stock solution of each compound. The daily standard working solutions, prepared at different concentration levels (from 0.01 ng g^−1^ to 900 ng g^−1^), were obtained by diluting the stock solution. All solutions were stored in amber containers at −18 °C until use. The laboratory apparatus used included an ultrasonic water bath (Test lab TB-10TA, Buenos Aires, Argentina), a vortex (ArcanoHX-2000-1, Buenos Aires, Argentina), and a centrifuge (U-320R-BOECO, Hamburg, Germany).

### 2.2. Instrumentation

The chromatographic conditions and mass spectrometric instrumental parameters were similar, as reported in previous work from our group [33]. The equipment used for the ultra-high-performance liquid chromatography analyses was an AcquityTM Ultra High-Performance LC system (Waters, Milford, CT, USA) equipped with an automatic sampler for sample injection and a binary pump system for solvent handling. The injection needle was washed with an appropriate mixture of acetonitrile and methanol prior to sample injection to avoid sample-to-sample contamination effects. Chromatographic separation was performed by injecting a sample volume of 10 μL into an ACQUITY UPLC^®^ BEH Phenyl (Waters, Milford, CT, USA) 2.1 mm internal diameter × 100 mm length and 1.7 μm particle size reversed-phase analytical column. Samples were run in a solvent gradient mode, changing the composition and flow rate of the mixture composed by aqueous solvent (A, water with 0.1% formic acid) and the organic solvent (B, acetonitrile with 0.1% formic acid) as follows (min, mL min^−1^): 50:50 (0.0, 0.25); 10:90 (3.0, 0.25); 0:100 (3.7, 0.2); 0:100 (4.8, 0.15); 50:50 (5.0, 0.15), and finally, 50:50 (5.5, 0.25). The temperature of the autosampler, including the sample vial carrier tray, was maintained at 4 °C, and the temperature of the column was kept constant at 30 °C. 

Mass spectrometric analysis was performed on a Quattro PremierTM XE Micromass MS Technologies instrument with a triple quadrupole analyzer that was equipped with an atmospheric pressure chemical ionization (APCI) source (Waters, Milford, CT, USA). The APCI ionization source, in positive polarity mode, provided better performance and, therefore, higher sensitivity in the detection of the analytes under study. Detection was performed in multiple reaction monitoring (MRM) modes of the selected ions in the first quadrupole (Q1) and third quadrupole (Q3). To select fragment ions of *m*/*z* (Q1) → *m*/*z* (Q3), a solution of the standard in acetonitrile was injected directly (via injection pump) into the spectrometer. Table S2 summarizes the optimal conditions for each analyte in the MRM mode. In order to favor the production of charged ions, infusion solutions of each analyte were prepared in acetonitrile with 0.1% (*v*/*v*) formic acid, which showed a higher ionization efficiency, and, consequently, a higher signal intensity was observed for most of the analytes. The APCI source was operated in positive mode at 400 °C with N_2_ as a nebulizer, and the temperature of the source was maintained at 120 °C. The current applied to the corona pin was set to 3 μA, the voltage of the extractor cone was set to 4 V, the desolvation gas flow rate (N_2_) was set to 200 L h^−1^, and the collision gas flow rate (Ar) was set to 0.18 mL min^−1^. The data obtained were processed using the instrumental software MassLynx Mass Spectrometry Software version 4.1 (Waters, Milford, CT, USA).

Chromatograms and mass spectra of product ions for NPAH and OPAH compound A are shown in Figure S1.

### 2.3. Sampling and Sample Preparation

All bee honey samples were identified and kept to avoid light (aluminum foil wrapped), humidity, heat, or cold to prevent the product’s deterioration and degradation of the analytes. The honey samples came from the provinces of San Luis, Corrientes, and Misiones (Argentina). Honey jars were bought from local commerce and artisan honey producers. Samples were named considering the location, GPS coordinates, and process as follows: ES-A, Corrientes, 30°00′58.6″ S 59°30′48.4″ W, handcrafted; ES-B, Corrientes, 30°00′58.6″ S 59°30′48.4″ W, handcrafted; M-A, Misiones; 27°36′11.8″ S 55°19′25.3″ W, commercial; SL-A, San Luis, 33°19′08.2″ S 66°19′50.9″ W, commercial; SL-B, San Luis, 32°37′20.8″ S 64°59′06.4″ W, handcrafted; SL-C, San Luis, 33°05′56.5″ S 65°52′51.0″ W, organic; and, SL-D, San Luis, 32°55′12.3″ S 66°18′15.2″ W, handcrafted. It is important to mention that in terms of sample classification, the term “Commercial” honey samples include those that undergo a high level of processing, from the collection using smoke or chemicals to their accumulation in cooperatives or companies that blend them with other kinds of honey from a different origin, homogenize them, add water content or, sometimes, even incorporate some additives to stabilize them on a large scale, either for local consumption and/or for export purposes.

On the other hand, the samples named “Handcrafted” refers to those that follow the typical beekeeping process, using smoke to repel insects, manual collection by dragging or sweeping over the hive, and decanting into containers for subsequent fractionation and marketing. Finally, “Organic” samples are understood as those collected utilizing bee removal techniques without the application of smoke or by the extraction of the queen bee from the hive for the natural migration of the swarm to another apiary far away from which it will be harvested. To optimize the extraction of NPAH and OPAH, honey types, formulations, composition, and production were considered. Laboratory samples were prepared with equal parts of each honey into an aqueous solution. In the same way, spiked samples were prepared with mixed solutions of known concentrations of NPAH and OPAH.

### 2.4. SALLE Procedure

**Figure 1:**
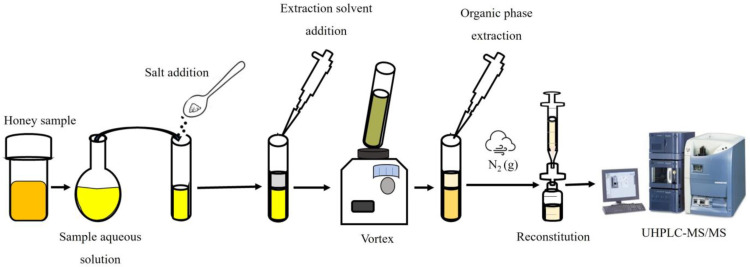
Schematic diagram of the liquid-liquid extraction assisted by salt saturation (SALLE) procedure.

The schematic procedure for the salting-out assisted liquid/liquid extraction with determination by liquid chromatography-tandem mass spectrometry is shown in Figure 1. An aliquot of 10 g honey was prepared in 30 mL ultrapure water. Then, the solution was thermostat and placed into a 50 mL tube with 6g of NaCl. Later, a 13.5 mL volume of acetonitrile, as extraction solvent, was added and stirred by vortex for 3 min. Subsequently, the mixture was centrifuged at 3500 rpm for 10 min. The organic phase was observed at the top of the tube, transferred into a glass tube, and brought to dryness with N_2_(g). Then, it was reconstituted into a 500 μL acetonitrile volume conditioned with 0.1% (*v*/*v*) formic acid and vortexed for 30 s. Finally, samples were filtered through 0.22 µm pore size filters and stored in amber vials until analysis by UHPLC-MS/MS.

### 2.5. Optimization and Experimental Design

It is important to understand the physicochemical characteristics of the compounds under study, such as the octanol-water partition coefficient (K_ow_) and the solubility in water (S). Although they present affinity for matrices with a high lipid content, NPAH and OPAH have a higher solubility than the original PAH and, therefore, present an improved capacity to be distributed between the aqueous phase and the organic phase. In virtue of this, the optimization of the variables related to the process of liquid extraction assisted by salt saturation was carried out using univariate and multivariate approaches. First, a screening of the variables that affected the extraction procedure was performed, then a full factorial design was applied for screening and optimization of the main experimental SALLE conditions, which were defined as (A) volume of water; (B) volume of extraction solvent; (C) salt concentration (%, *w*/*v*) and (D) ultrasound time (see Table S3 in the Supplementary Materials). In this screening stage, a two-level full factorial design 2^4^ was used. Sixteen experimental points and five central points were considered, resulting in 21 trials. The variables were evaluated at two levels, minimum (denoted as −1) and maximum (denoted as +1), and the central points, with five replicates (level 0), to estimate the experimental variance and verify the loss of linearity between the levels chosen for each variable (curvature verification). The statistical significance of each factor and its combination were evaluated at the significance level (α) of 5%. In all cases, the maximum possible extraction recovery (%) was pursued; thus, each analyte chromatographic peak average area was considered the response. 

Once the most significant variables were selected, a second full factorial design was carried out. The variables for this trial were: (A) volume of water and (B) volume of extraction solvent. Their values were optimized through a central composite design (CCD) based on the response surface design (RSM) method (Table S4). The CCD design consisted of four axial points, four factorial, and three central points, with a total number of eleven trials, which were conducted randomly. Again, two levels were used together with a central point for the variables (A) volume of water and (B) volume of extraction solvent.

### 2.6. Method Validation

#### 2.6.1. Matrix Effect

Matrix effect (*ME*(%)) is a well-known significant trouble in applications that focus on mass spectrometry, mainly when ion sources probably affect the stability of different compounds and/or due to the extensive constituents present in the sample; among others; that could modify the response of the analytes through suppression or enhancement of the signal, reporting erroneous results [34]. A disadvantage of coupling an atmospheric pressure ionization source, such as APCI coupled to mass spectrometric detectors, is that the ionization is susceptible to signal suppression/exaltation by the sample matrix [34]. The effect of the sample matrix is evaluated by comparing the spiked samples’ signal with the spiked pure solvent’s signal, as followed in this work. As an indicator of the magnitude of ion suppression or signal enhancement, the percentage of the ratio of the slopes in the spiked samples and calibration solutions was used. The matrix effect was calculated as follows:

(1) $$ME\;(\%)=[100-(\left(\frac{Slope\;(spiked\;sample)}{Slope\;(calibration\;solutions)}\right)\times 100]$$

#### 2.6.2. Analytical Capacity

A defined mass of all the samples collected, previously homogenized, was considered to optimize the variables that affect the extraction of the compounds under study. Thus, 10 g of honey in 30 mL of an aqueous solution was prepared. The results obtained during the optimization process were presented and compared by evaluating the Enrichment Factor (*EF*) and the Recovery factor (*R*), which were calculated as follows:

(2) $$EF=\frac{concentration\;in\;organic\;phase}{concentration\;in\;the\;aqueous\;phase}$$

(3) $$R\left(\%\right)=\left[\frac{\left(Quantity\;found-Actual\;quantity\right)}{\left(Quantity\;added\right)}\right]\times 100$$

To optimize the proposed extraction methodology and after considering the levels of some PAH in food samples [6], the samples were spiked with known concentrations of NPAH and OPAH from 0.01 ng g^−1^ to 900 ng g^−1^. This approach considered multiple calibration points, linearity, and normality. As a result, limits of detection (*LOD*) and quantification (*LOQ*) were calculated according to Equations (4) and (5) [35]. Where $\overset{-}{x}$ corresponds to the mean concentration; *S_y/x_*, the residual standard deviation; *b*, the slope of the calibration curve; *m*, the number of replicates per concentration level of the spiked samples; and *n*, the number of concentration levels for spiked samples: *i* = 1, 2, …, *I*.

(4) $$LOD=\frac{{3.3S}_{y/x}}{b}\sqrt{\frac{1}{m}+\frac{1}{n}+\frac{{\overset{-}{x}}^{2}}{\sum_{i=1}^{n}{\left(x_{i}-\overset{-}{x}\right)}^{2}}}$$

(5) $$LOQ=\frac{{10S}_{y/x}}{b}\sqrt{\frac{1}{m}+\frac{1}{n}+\frac{{\overset{-}{x}}^{2}}{\sum_{i=1}^{n}{\left(x_{i}-\overset{-}{x}\right)}^{2}}}$$

### 2.7. Sustainability Assessment 

The Green Certificate evaluation was used to assess the impact of the proposed procedure, the nature and quantity of reagents used, and the amount of waste generated. This indicator is based on the calculation of a score, with 100 representing the ideal green analysis (with its maximum value corresponding to outcome *A*, as the greenest method) and the opposite being red (outcome G, indicating a method that is not sustainable). Penalty points (PPs) are assigned on a sliding scale if the process has hazardous chemical use, waste generation, occupational exposure risk, or high energy consumption. The higher the Green Certificate score, the greener the analytical process. Equations used to calculate the penalty points are as follows [36]:

(6) $$PPR=\left(0.61\pm 0.05\right)V^{\left(0.31\pm 0.02\right)}$$

(7) $$PPW=\left(1.50\pm 0.08\right)W^{\left(0.4\pm 0.02\right)}$$

where *PPR* is penalty points for reagents, *PPW* is penalty points for waste generated, *V* is the volume of solvent generated, and *W* is the volume of waste. Penalty Points are based on the Eco-Analytical Scale, and its considerations are shown in Table S5.

## 3. Results

### 3.1. Optimization SALLE

#### 3.1.1. Univariate Optimization 

First, the extraction solvent was chosen, considering the physic-chemical properties of the compounds under study. The recovery efficiency in the extraction (R (%)) using the following organic solvents was evaluated: acetonitrile (δ: 0.78 g mL^−1^), hexane (δ: 0.66 g mL^−1^), toluene (δ: 0.87 g mL^−1^), dichloromethane (δ: 1.33 g mL^−1^), and chloroform (δ: 1.44 g mL^−1^). Results show that acetonitrile was better than other solvents in the general conditions (Figure S2A). It is important to mention that for dichloromethane and chloroform, no differentiation of the aqueous and organic phases was observed due to the formation of an emulsified and precipitated phase between the matrix components, making it impossible to recover the organic phase. Second, with the selection of the nature of the extraction solvent, the effect of salting out on the extraction efficiency of the compounds was evaluated. In this context, the following salts were used: sodium chloride (NaCl), potassium chloride (KCl), magnesium sulfate (MgSO_4_), and ammonium sulfate ((NH_4_)_2_SO_4_). From the obtained recoveries, it was possible to observe an improvement in the separation of the aqueous phase and the various organic solvents, in addition to a recovery value at significantly higher salt concentrations when used together with acetonitrile. In this sense, salt masses were evaluated from 1 to 6 g (20 % (*w*/*v*), optimized previously by the experimental design). In all cases, satisfactory recoveries were observed (Figure S2B). In this context, due to the low cost and better differentiation of phases, NaCl was selected for the salting-out stage of the proposed methodology. Third, the effect of pH is of paramount importance in SALLE. It is often necessary to make pH modifications to achieve acid-base partitioning of analytes and ensure that most are found in their neutral form to be efficiently transferred from the aqueous phase to the organic phase. In this sense, the effect of pH on the sample solutions was studied. For this, different additives and buffer solutions compatible with the separation/determination system were used to conditionate the pH in the samples at the following values: 3 (formic acid), 5 (acetic acid), 7 (sodium bicarbonate), and 9 (ammonium hydroxide). Since a pH equal to 7 agreed with the pH of the honey samples and no significant differences between the pH values were observed on the analytes’ recoveries, this pH value was selected (Figure S2C). 

Univariate parameter optimization continued, sample mass (0.5, 1, 3, 5, 7.5, and 10 g of honey), vortex time (0.5, 1, 2, 3, and 4 min), and the subsequent efficient phase separation (centrifugation time, 1 to 10 min and from 2000 to 3500 rpm) were evaluated to favor the extraction of analytes of interest.

Overall, no changes in analytical yields were observed when a 10 g of sample, a vortex time of 3 min, and a centrifugation step of 10 min at 3500 rpm were chosen (Figure S3).

#### 3.1.2. Full Factorial Design

The experimental design is a critical optimization approach because it allows the study of many factors that can co-occur and their interactions, which could affect the recovery of compounds during the extraction process. After its application, as mentioned in Section 2.5, the significant main effects on analytes response were observed to be the following variables: the volume of water (A), the volume of acetonitrile (B), and the interaction between them (AB); meanwhile, NaCl concentration (C) and ultrasound assistance time (D) were not significant; these findings can be observed in the Pareto graph (Figure S4). Normal probability plots also allowed the verification of the conditions mentioned above.

#### 3.1.3. Response Surface Methodology (RSM) and Composite Central Design (DCC)

A central composite design, based on the response surface design (RSM) methodology, was applied from the obtained results. The chromatographic peak area of each analyte was used to estimate if there is a curvature in the region of the experimental space and provide improvements in the methodology. The analysis of variance (ANOVA) and regression were used to evaluate the importance of the variables and their interactions, which were calculated with the Design Expert software 8.0.0 (Stat-Ease, Inc., Minneapolis, USA) ^®^.

**Figure 2:**
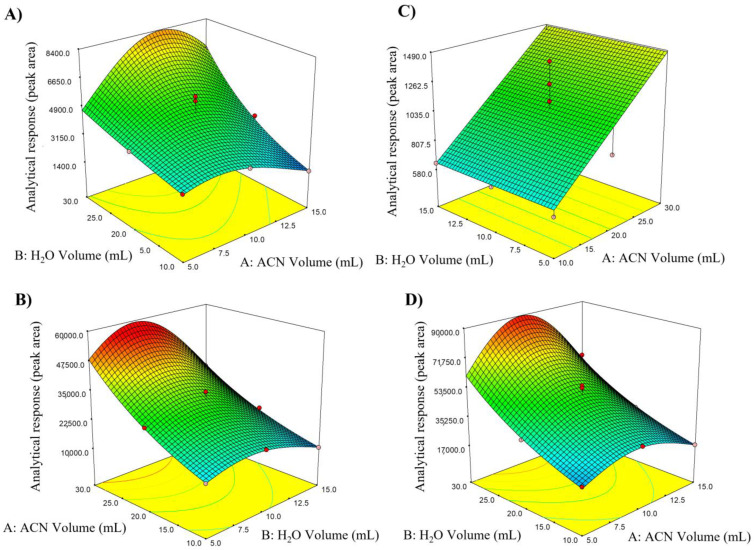
Surface plots for optimization of the acetonitrile and water volumes through Central Composite Design based on the response surface design methodology. Model compounds: (**A**) 1-NPYR; (**B**) 5,12-NAPHTONA; (**C**) 2-NFLU y; (**D**) 2-FLUCHO.

From the analysis of ANOVA for each analyte, the model was demonstrated to be significant, *p*-value < 0.0001, in all cases. Additionally, the *p*-value of non-adjustment (LOF) was not significant, which evidenced the suitability of the selected model. The suitability of each model was also assessed by considering the coefficients of determination (R^2^) and the adjusted coefficients of determination (R^2^_aj_). Thus, a good coefficient of determination (R^2^ ≥ 0.90) was obtained, indicating good agreement and adjustability with the experimental data. Similarly, an R^2^_aj_ > 0.90 indicated the goodness of the approach to function as a predictive tool. A summary of these conditions is shown in Table S6. After analyzing the model equations for all analytes, it was generally observed that the two main factors (A and B) and their second-order interactions have an important influence on extraction yields. Thus, three-dimensional graphs of the model were used to visualize each factor’s effect on the response. For example, in Figure 2, the surface plots for some of the NPAH and OPAH are shown, and similar graphs were obtained for the rest of the analytes. Subsequently, it was necessary to recognize the surface zone where the specifications for the proposed methodology were maximized, for this purpose, the desirability function. The desirability function was used to predict optimal experimental conditions, which has implications for modifying each expected response variable to a desirability value ranging from zero (0, undesirable response) to one (1, optimal response). After optimization, the experimental conditions (maximum in the desirability ratio (D = 0.998)) were: acetonitrile volume: 13.5 mL and water volume: 30 mL (Figure S5). These optimal conditions suggested by the statistical model were experimentally corroborated, and a good agreement with the results was obtained.

### 3.2. Methodological Validation

Samples were spiked at varied concentration levels in triplicates (*n* = 3). These samples were analyzed to establish the recoveries and linearity through the calibration curves. Recovery experiments were conducted using individual and pooled honey samples, spiked with the analytes at concentrations from 0.01 to 300 ng g^−1^ for 1-NPYR; 3-NFLUANTH and concentrations from 0.01 to 900 ng g^−1^ for 2-NFLU; 9-NANTHR; 5,12-NAPHTONE; 9,10-ANTHRONA; and 2-FLUCHO.

LOD and LOQ were calculated according to Equations (4) and (5). Calibration curves were obtained by linear regression of least squares of signal intensity vs. the concentration of each NPAH and OPAH. Good linearity was observed, with all analytes’ correlation coefficients (r^2^) greater than 0.98. In addition, the F-test was applied, and linear regression was statistically acceptable at a 95% confidence level. The method’s reproducibility was assessed using the relative standard deviation (RSD, *n* = 3).

Additionally, EF values between 53 and 60 times were obtained, and recoveries ranged from 90.6 to 100.1%, much better than those already reported for PAH. A summary of the analytical performance of the proposed methodology in honey samples is presented in Table 1. From the findings, LOD, LOQ, and EF were similar to those reported by other authors in the determination of non-substituted PAH in this type of matrices [37,38,39,40], which is the closest report related to compounds of this nature available so far in the literature. Regarding the evaluation of the matrix effect, as mentioned in Section 2.6.1, the percentage of the quotient of the slopes in the spiked samples and calibration solutions was used as an indicator of its extent. No signal enhancement, but a response average reduction of approximately 95% for all the analytes under study was observed due to the honey matrix interference. This effect drastically diminished after the SALLE extraction; thus, the signal obtained for each analyte was not affected by matrix suppression. Therefore, quantification was performed through external calibration.

### 3.3. Greenness Assessment

In this work, the score obtained for the proposed SALLE-UHPLC-MS/MS methodology was 86.8 (~87 points), corresponding to a satisfactory result. The Green Certificate metric was applied to evaluate the impact of the procedure on the environment [36]. Details of this metric application are shown in Table 2; as can be seen, excellent sustainability was demonstrated.

## 4. Discussion

The proposed SALLE-UHPLC-MS/MS methodology is sufficient, simple, robust, and applicable to routine analysis. As mentioned, food intake is the first route of contamination with these toxic compounds in non-smokers since they can generate in food during storage, transport, or cooking processes (mainly grilled, roasted, smoked, and fried). In addition, contamination during the washing and reception of raw materials, growth of crops on contaminated soils, incineration of agricultural residues, and burning of biomass, along with the deposition of air particles in food during post-harvest and food processing phases contribute to the presence of PAH and their derivatives in these samples. 

Honey samples, commercial, handcrafted, and organic honey samples were analyzed by the optimal methodology. The results are shown in Table 3. The presence of PAH derivatives was found in the real samples at concentration levels from 5.0 to 404.9 ng g^−1^ for NPAH and from 1.7 to 624.9 ng g^−1^ for OPAH. On the other hand, 9-NANTHR and 9,10-ANTHRONA were not determined in any sample. The found concentrations were higher than those reported in the literature for their predecessor’s PAH, indicating that NPAH and OPAH are more abundant in aqueous matrices, such as honey, possibly due to the higher solubility in water of these compounds compared to their unsubstituted molecules. No anthracene derivatives (such as 9-NANTHR and 9,10-ANTHRONA) were detected. This may be due to this compound’s low stability in various matrices due to its thermal and/or light decomposition [37,39,40,41].

It is known that the presence of 1-NPYR and 3-NFLUANTH in environmental samples is an indicator of anthropogenic contamination [42]. In this sense, the samples collected near urban areas (SL-B and SL-D) and those from commercial origin (M-A) presented the highest concentrations of these compounds, possibly because the sampling sites might be influenced by vehicular traffic and residential emission sources. On the other hand, it was possible to observe that most of the NPAH and OPAH studied (except by 3-NFLUANTH) were not detected in the honey samples named SL-C, which was collected applying more organic techniques, such as bee sweeping, dry hitting, the use of air, or moving the queen bee away from the hive. In addition, these results may be due to the collection of these honey samples in an area with the scarce influence of anthropogenic emission sources. However, in all the analyzed samples, similar concentrations of 3-NFLUANTH were found at levels from 24.8 to 41.7 ng g^−1^. 

From the findings, it is possible to infer the presence of nitrated and oxygenated PAH derivatives, which originate mainly due to anthropogenic emissions, since the samples were collected near urban areas, high-traffic areas, lakes, and contaminated soils in the vicinity of apiaries. Finally, the concentration levels of the PAH derivatives reported demonstrated the high toxicity and distribution in the environment of these contaminants, which points out the importance and need for their continuous assessment in food samples.

## 5. Conclusions

A methodology for the efficient extraction, preconcentration, and simultaneous determination of NPAH and OPAH in honey samples based on the SALLE-UHPLC-MS/MS is reported for the first time. The proposed methodology has characteristics of simplicity, accuracy, precision, and sustainability, and it can be successfully applied to routine analysis, quality assurance, and food safety protocols. From the findings in real samples, levels of the majority of the studied NPAH and OPAH were determined. The compounds 9-NANTHR and 9,10-ANTHRONA were not detected, probably due to the light and thermal stability of their predecessor. The concentrations found varied from 1.7 to 624.9 ng g^−1^, which resulted in being higher than those reported for unsubstituted PAH in honey samples, probably due to the solubility of the PAH derivatives. Likewise, the highest concentrations of NPAH and OPAH corresponded to samples collected near urban areas and manufactured samples. In contrast, in samples from organic processing, NPAH and OPAH were not detected, except for 3-NFLUANTH, which was found in all samples. These findings are estimated to be related to emissions from fossil fuel combustion and atmospheric reactions of PAH derivatization.

## Figures and Tables

**Table 1:** Analytical figures of merit for the developed methodology.

Compounds	*r* ^2^	Linear Range (ng g^−1^)	LOD (ng g^−1^)	LOQ (ng g^−1^)	EF (folds)	R (%)	Precision (RSD%) *n* = 3
***NPAH***							
1-NPYR	0.998	1.6–150.0	0.54	1.64	55	92.0	6.40
2-NFLU	0.992	2.1–500.0	0.70	2.10	57	95.7	8.90
3-NFLUANTH	0.989	0.8–150.0	0.26	0.79	59	99.4	1.90
9-NANTHR	0.986	12.5–300.0	7.42	12.49	60	100.1	5.40
***OPAH***							
5,12-NAPHTONA	0.974	0.1–500.0	0.04	0.12	53	90.6	6.01
9,10-ANTHRONA	0.993	20.4–300.0	9.77	20.36	57	95.9	6.80
2-FLUCHO	0.998	0.3–750.0	0.09	0.27	55	92.6	7.90

**Table 2:** Green certificate assessment for the proposed liquid–liquid extraction assisted by salt saturation (SALLE) methodology.

Reagents Penalty Points (PP_R_)				Energy (PP_E_)	Risk (Occupational Hazard)	Waste (PPW)	Total (100-PP)	Green Certificate *	Green Metric Score
Regents Type	Regent Amount (mL)—(g)	Hazards	Subtotal						
Acetonitrile	13.5 mL	1.6	6.2	2 (>1.5 kWh/sample)	0	5	13.2	B	86.8
NaCl	6 g	1	0						

* The final assessment relates to a letter (from A to G) and a color range (from dark green (A) to red (G)).

**Table 3:** Found concentrations of NPAH and OPAH in honey samples.

Compounds	Concentrations Determined in Honey Samples (ng g ^−1^) ^a^						
	ES-A (Handcrafted)	ES-B (Handcrafted)	SL-A (Commercial)	SL-B (Handcrafted)	SL-C (Organic)	SL-D (Handcrafted)	M-A (Commercial)
1-NPYR	N.D. ^b.^	^b^	5 ± 1	9 ± 1	^b^	21 ± 1	23 ± 2
2-NFLU	^b^	^b^	82 ± 1	152 ± 0.1	^b^	405 ± 1	398 ± 10
3-NFLUANTH	42 ± 2	25 ± 2	36 ± 6	39 ± 4	26 ± 2	31 ± 1	33 ± 5
9-NANTHR	^b^	^b^	^b^	^b^	^b^	^b^	^b^
5,12-NAPHTONA	300 ± 32	305 ± 7	194 ± 22	162 ± 10	^b^	183 ± 10	190 ± 10
9,10-ANTHRONA	^b^	^b^	^b^	^b^	^b^	^b^	^b^
2-FLUCHO	625 ± 51	287 ± 4	104 ± 9	587 ± 78	2.0 ± 0.1	487 ± 27	270 ± 20

^a^ Mean value ± standard deviation (*n* = 3); ^b^ N.D.: Not Detected.

## Data Availability

Data is contained within the article or supplementary material.

## Supplementary Materials

The following supporting information can be downloaded at: https://www.mdpi.com/article/10.3390/foods12112205/s1, Table S1. Structures of environmentally relevant nitrated and oxygenated polycyclic aromatic hydrocarbons; Table S2. Optimized MRM scanning mode mass spectrometer conditions for NPAH and OPAH; Table S3. Variables and levels used in the 2^4^ Full Factorial Design; Table S4. Variables and levels used in the Central Composite Design; Table S5. Penalty Points (PPs) considering the Analytical Eco-Scale; Table S6. ANOVA chart of statistical effects; Figure S1. Chromatograms and mass spectra of products ions for NPAH and OPAH compounds for UHPLC-(+)APCI-MS/MS optimal conditions; Figure S2. Optimization of the nature of the solvent (A), ionic strength effects (B), and pH (C) for the SALLE procedure; Figure S3. Optimization of vortex agitation (A), centrifugation time (B), and mass sample (C) for the SALLE procedure; Figure S4. Pareto Chart of the main effects in the SALLE procedure for the simultaneous extraction of NPAHs and OPAHs; Figure S5. Representation of the desirability function for the optimized variables.
