# Supplementary material for: Evaluation of Environmentally Relevant Nitrated and Oxygenated Polycyclic Aromatic Hydrocarbons in Honey
Source: Foods. 2023 May 31;12(11):2205. doi: 10.3390/foods12112205 (PMC10252447; doi:10.3390/foods12112205)
Supplement: Supplementary file 1 [file foods-12-02205-s001.zip › foods-2382342-supplementary.pdf]

Supplementary Material

Evaluation of environmentally relevant nitrated and oxygenated polycyclic aromatic hydrocarbons in honey

Alejandro Mandelli [a,b], María Guiñez [a,b*,] Soledad Cerutti [a,b*]

[a] *Instituto de Química de San Luis (INQUISAL, UNSL-CONICET CCT-San Luis), Área de Química Analítica, Facultad de Química Bioquímica y Farmacia, Universidad Nacional de San Luis, Laboratorio de Espectrometría de Masas, Bloque III, Ejército de los Andes 950, San Luis, CP 5700, Argentina.*

[b] *Consejo Nacional de Investigaciones Científicas y Técnicas (CONICET), Godoy Cruz 2290, C1425FQB Buenos Aires, Argentina.*

*Corresponding authors: Tel: +54 0266 4520300*1311.

Table S1. Structures of environmentally relevant nitrated and oxygenated polycyclic aromatic hydrocarbons.

**Compound**	**Empirical formula**	**Structure**	**Abbreviation**	**Molecular Weight ($g\ mol^{-1}$)**
1-Nitropyrene	$C_{16}H_9NO_2$	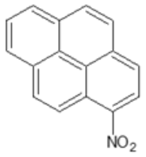	1-NPYR	247.25
2-Nitrofluorene	$C_{13}H_9NO_2$	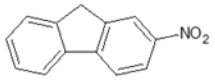	2-NFLU	211.22
3-Nitrofluoranthene	$C_{16}H_9NO_2$	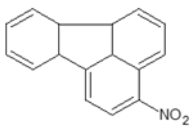	3-NFLUANT	247.25
9-Nitroanthracene	$C_{14}H_9NO_2$	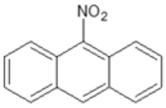	9-NANTHR	223.23
5,12-Naphthacenequinone	$C_{18}H_{10}O_2$	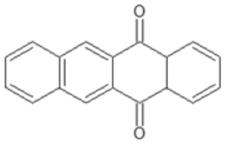	5,12-NAPHTONA	258.27
9,10-Anthracenequinone	$C_{14}H_8O_2$	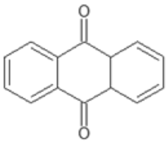	9,10-ANTHRONA	208.21
2-Fluorenecarboxaldehyde	$C_{14}H_{10}O$	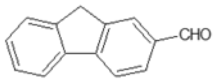	2-FLUCHO	194.23

Table S2. Optimized MRM scanning mode mass spectrometer conditions for NPAH and OPAH.

Compound	Cone (V)	Precursor Ion (m/z)	Collision (V)	Product Ion (m/z)
NPAH				
**1-NPYR**	30	248	16<br>25<br>30	218<br>202*<br>190
**2-NFLU**	30	212	12<br>17	195<br>165*
**3-NFLUANTH**	19	248	17<br>16<br>20	231*<br>218<br>190
**9-NANTHR**	19	224	8<br>30	207<br>178*
OPAH				
**5,12-NAPHTONA**	10	259	35<br>21<br>23	242<br>231<br>203*
**9,10-ANTHRONA**	35	209	20<br>20	181<br>153*
**2-FLUCHO**	32	195	16	167*

* Transition selected for quantification

Table S3. Variables and levels used in the 2^4 Full Factorial Design.

Factor	Variable	Levels		
		**-1**	**0**	**+1**
**A**	Acetonitrile volume (mL)	1	5	9
**B**	Water volume (mL)	5	10	15
**C**	NaCl concentration (%, w/v)	20	35	50
**D**	Ultrasound-assisted agitation time (min)	0	15	30

Table S4. Variables and levels used in the Central Composite Design.

Factor	Variable	Levels		
		**-1**	**0**	**+1**
**A**	Acetonitrile volume (mL)	5	10	15
**B**	Water volume (mL)	10	20	30

Table S5. Penalty Points (PPs) considering the Analytical Eco-Scale.

Category	Sub-total PPs		Total PPs
	**Reagents**		
**Amount**	<10 mL (<10 g)	1	PPs quantity x PPs denomination
	10-100 mL (10-100 g)	2	
	>100 mL (>100 g)	3	
**Hazards**	Nonexistent	0	
	Mild	1	
	High	2	
	**Instrument**		
**Energy**	<0.1 kWh per sample		0
	<1.5 kWh per sample		1
	>1.5 kWh per sample		2
**Risk**	No emission of vapors into the atmosphere		0
	Emission of vapors into the atmosphere		3
**Waste**	Nonexistent		0
	<1 mL (<1 g)		1
	1-10 mL (1-10 g)		3
	>10 mL (>10 g)		5
	Recycled		0
	Degradation		1
	Inactivate		2
	No treatment		3

Table S6. ANOVA chart of statistical effects.

Compound	*Response Transformation*	*Modeling*	p^{a}-value		$R^2_{adj.}$	*CV (%)*
			Modeling	*Lack of adjustment*		
**1-NPYR**	Natural Logarithm	Quadratic	0.0062	0.9045	0.9073	1.7
**2-NFLU**	Base 10 Logarithm	Linear	0.0068	0.9140	0.9585	1.8
**3-NFLUANTH**	Natural Logarithm	Quadratic	< 0.0001	0.9484	0.9935	0.4
**9-NANTHR**	Natural Logarithm	Linear	< 0.0001	0.0818	0.9493	1.5
**5.12-NAPHTONE**	Natural Logarithm	Quadratic	< 0.0001	0.9022	0.9805	0.6
**9.10-ANTHRONE**	Natural Logarithm	Quadratic	< 0.0001	0.8949	0.9905	0.5
**2-FLUCHO**	Natural Logarithm	Quadratic	< 0.0001	0.8523	0.9801	0.6

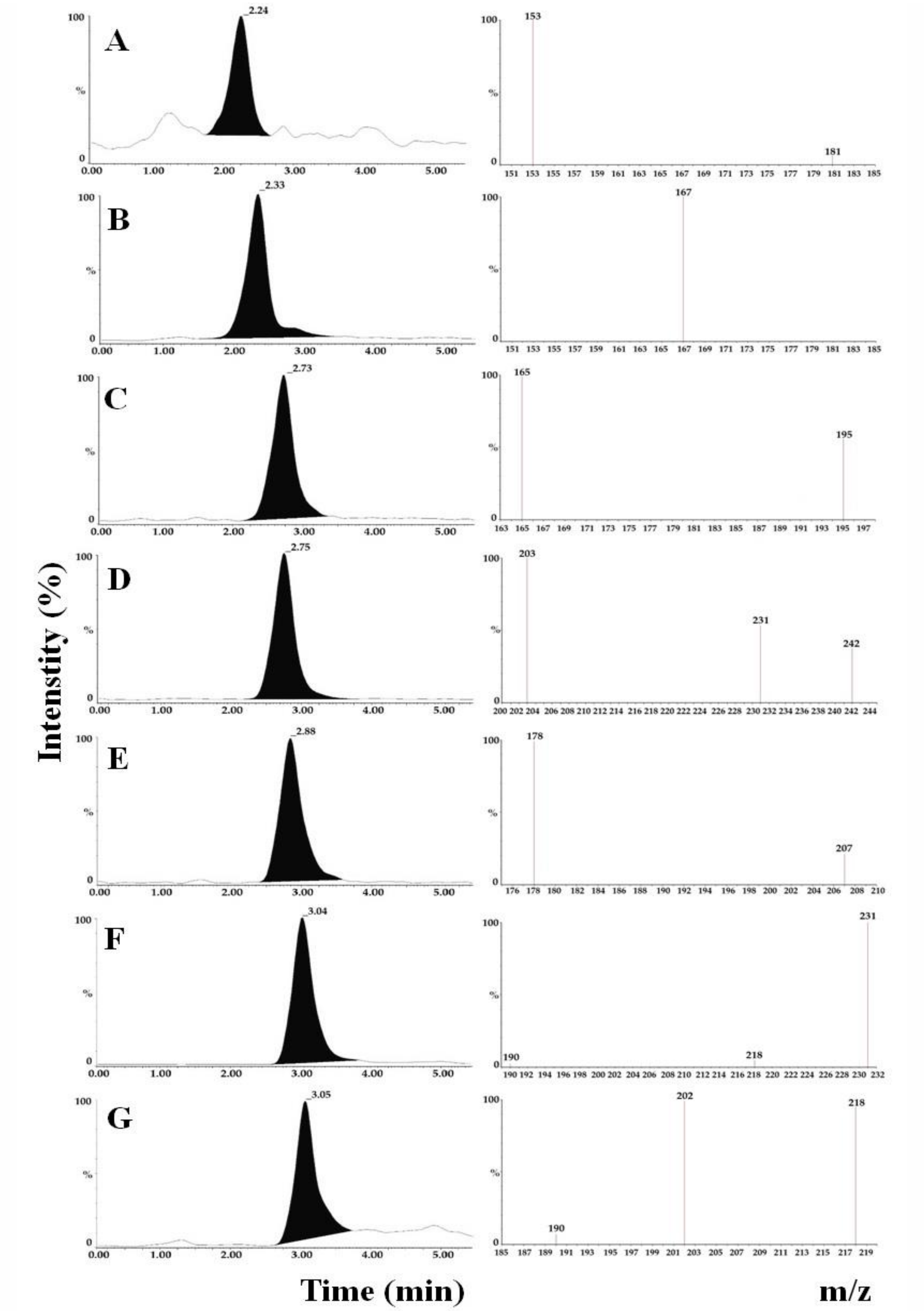

Figure S1. Chromatograms and mass spectra of products ions for NPAH and OPAH compounds for UHPLC- (+)APCI-MS/MS optimal conditions, (A) 9,10-ANTHRONA (Retention time, Rt: 2.24 min); (B) 2-FLUCHO (Rt: 2.36 min); (C) 2-NFLU (Rt: 2.73 min); (D) 5,12-NAPHTONE (Rt: 2.75 min); (E) 9-NANTHR (Rt: 2.88 min); (F) 3-NFLUANTH (Rt: 3.04 min), and (G) 1-NPYR (Rt: 3.05 min).

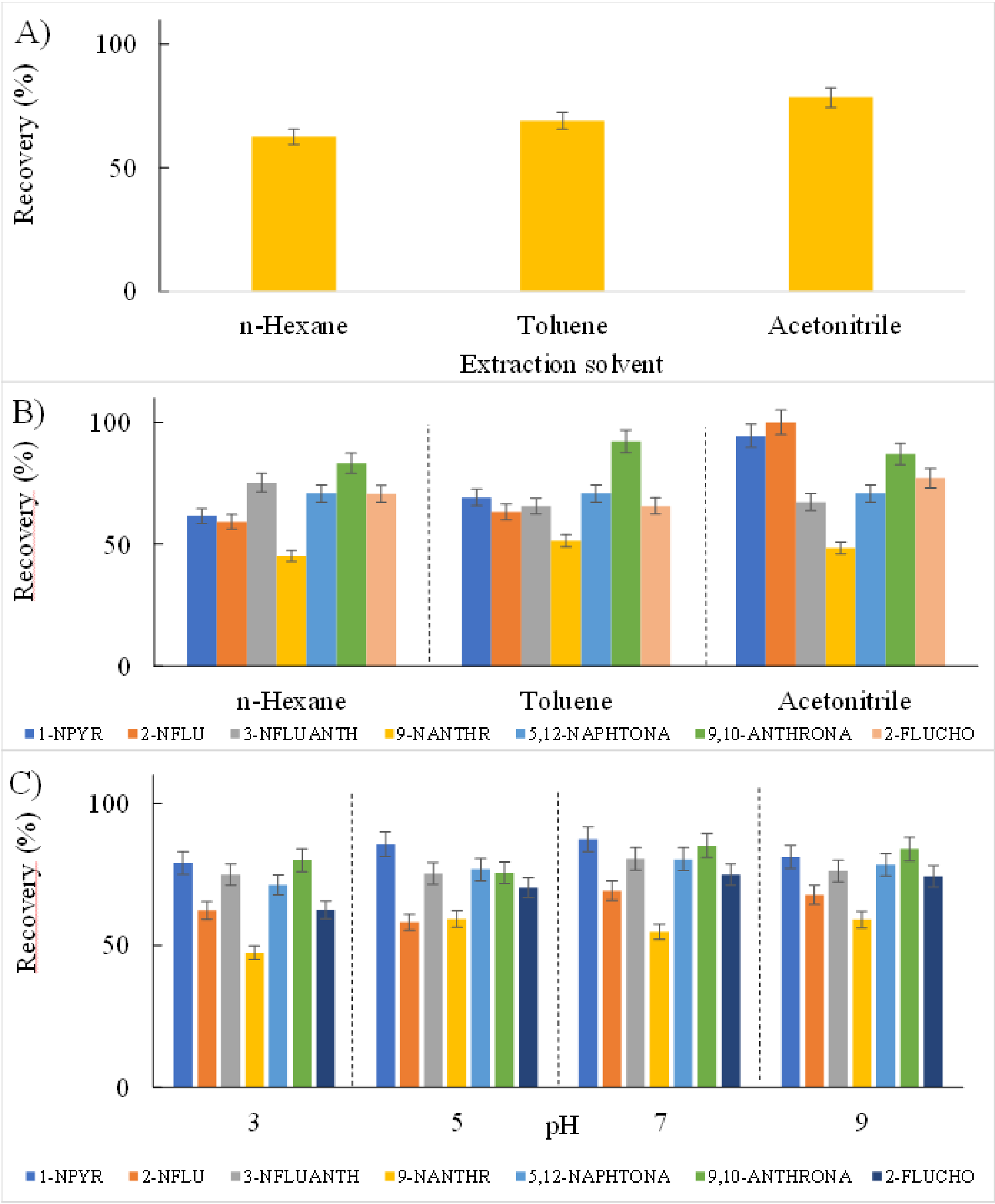

Figure S2. Optimization of the nature of the solvent (A), ionic strength effects (B), and pH (C) for the SALLE procedure.

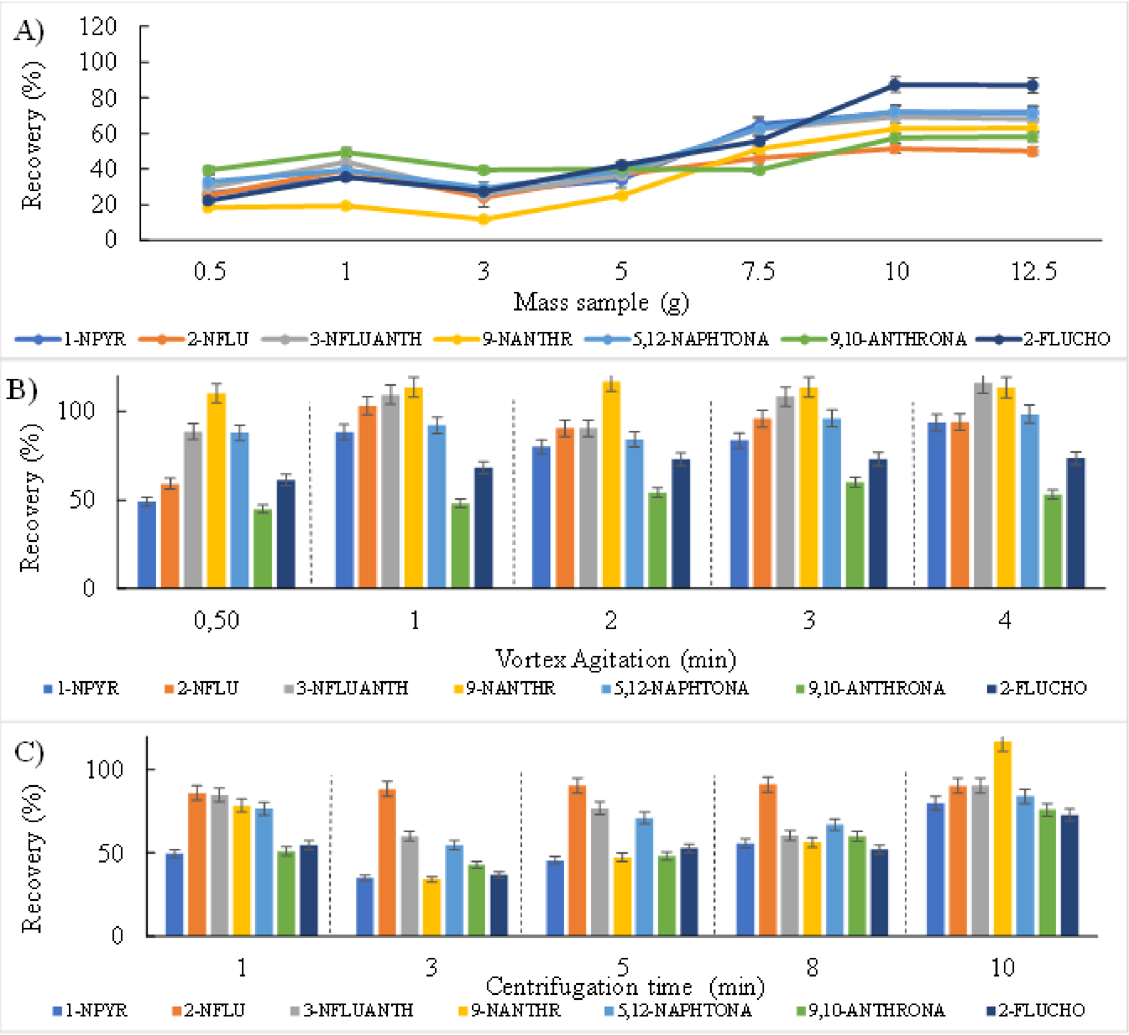

Figure S3. Optimization of vortex agitation (**A**), centrifugation time (**B**), and mass sample (**C**) for the SALLE procedure.

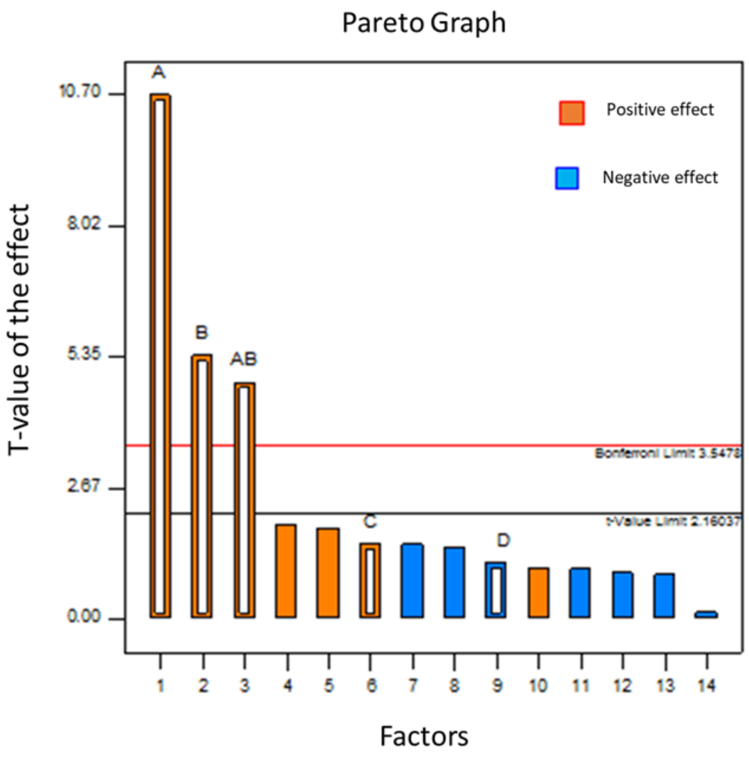

Figure S4. Pareto Chart of the main effects in the SALLE procedure for the simultaneous extraction of NPAHs and OPAHs. A: Volume of water. B: Volume of acetonitrile. AB: interaction between water and acetonitrile. C: NaCl concentration. D: Ultrasound extraction time

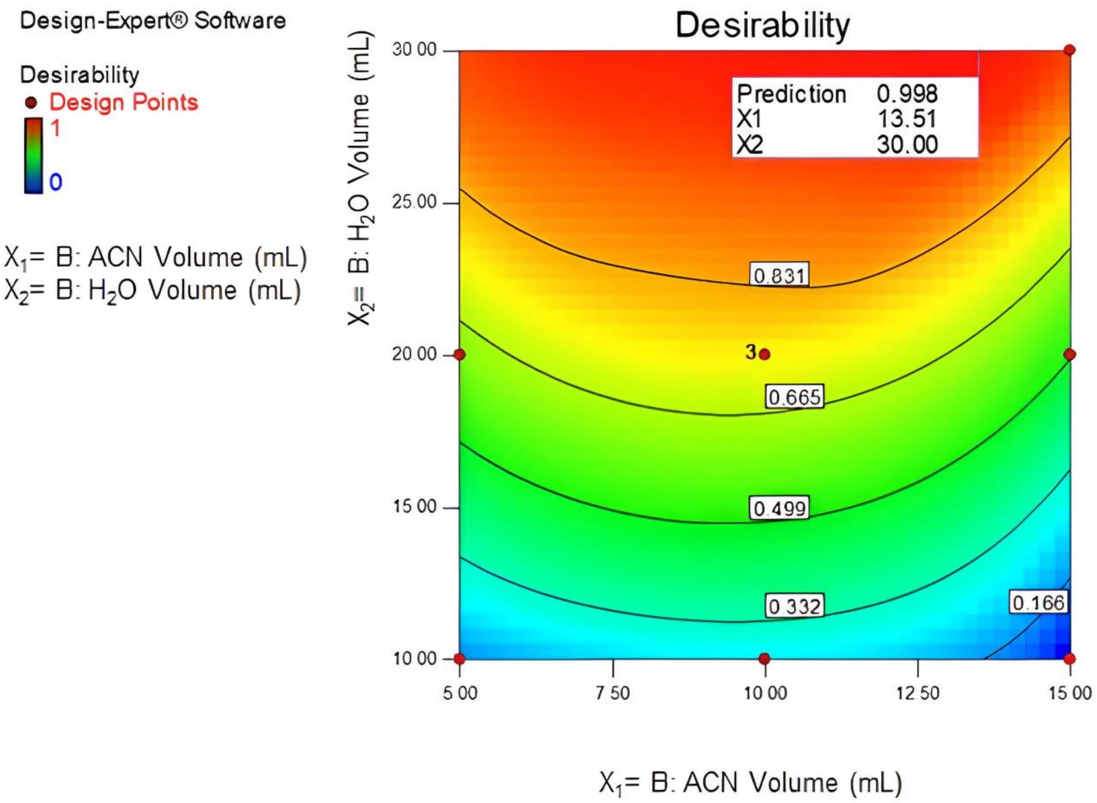

Figure S5. Representation of the desirability function for the optimized variables.
